# Advancements in imaging research in thyroid-associated ophthalmopathy

**DOI:** 10.3389/fendo.2025.1601556

**Published:** 2025-09-08

**Authors:** Zuxing Xu, Zhe Xue, Zhaohui Lyu

**Affiliations:** Department of Endocrinology, the First Medical Center of Chinese PLA General Hospital, Beijing, China

**Keywords:** thyroid-associated ophthalmopathy, ultrasonography, computed tomography, magnetic resonance imaging, clinical management

## Abstract

Thyroid-associated ophthalmopathy (TAO), a sight-threatening ocular condition intricately associated with autoimmune thyroid diseases, is the most common orbital disorder among adults. Accurate assessment of TAO is crucial for effective clinical management. However, the current evaluation system is hindered by significant subjectivity and a lack of standardized objective criteria, thereby complicating the pursuit of precise and individualized treatment strategies. Imaging techniques are integral to the clinical management of TAO, as they provide detailed anatomical visualization of the orbit and reflect underlying pathophysiological changes. This article reviews the applications of three prevalent imaging modalities—ultrasonography, computed tomography (CT), and magnetic resonance imaging (MRI)—in the diagnosis and management of TAO. We examine their respective advantages, limitations, and roles in disease diagnosis, staging, and evaluation of therapeutic efficacy, with the aim of providing a scientific basis for the optimization of clinical practice.

## 1 Introduction

Thyroid-associated ophthalmopathy (TAO) is an orbital disorder closely related to autoimmune thyroid diseases. The clinical presentation of TAO is multifaceted and varied, predominantly encompassing proptosis, eyelid retraction, and diplopia, among other symptoms. In severe instances, the condition may advance to dysthyroid optic neuropathy (DON). These manifestations not only alter patients’ physical appearance but also have detrimental psychological and social repercussions, thereby substantially diminishing their quality of life (1). A precise evaluation of the activity and severity of TAO is essential for formulating rational and effective therapeutic strategies.

Currently, the clinical evaluation of TAO predominantly employs assessments such as the Clinical Activity Score (CAS), NOSPECS severity classification, the European Group on Graves’ Orbitopathy (EUGOGO) severity classification, and the Graves’ Ophthalmopathy Quality of Life (GO-QoL) questionnaire for disease staging and grading (2). Nonetheless, these existing methodologies largely rely on subjective clinical symptoms and physical signs, which present certain limitations regarding accuracy and objectivity. Consequently, there is a pressing need for a more objective and precise assessment approach to enhance the diagnosis and management of TAO.

Imaging examinations offer a range of qualitative and quantitative indicators that can more objectively and comprehensively reflect alterations in the orbital structures and physiological conditions of TAO patients. While ultrasonography is primarily utilized to assess TAO by monitoring orbital hemodynamic changes, it is limited in its ability to visualize deeper structures, such as the extraocular muscles and the optic nerve. Computed tomography (CT) is excellent for measuring exophthalmometric values and the structure of extraocular muscles quantitatively. However, CT involves ionizing radiation and is not effective at visualizing soft tissue inflammation. On the other hand, magnetic resonance imaging (MRI) is a non-invasive method with high resolution for soft tissues, offering clear images of orbital structures. The multi-parametric imaging capabilities of MRI provide unique benefits for evaluating TAO. Imaging methods, especially MRI, are crucial for accurately assessing TAO, offering more dependable evidence for personalized diagnosis and treatment. The comparison of these techniques are summarized in **Table 1**. In the following, the applications of three imaging methods in TAO are reviewed in detail.

**Table 1:** Comparison of three imaging techniques.

Aspect	Ultrasound	CT	MRI
Imaging Focus	Hemodynamic changes	Orbital bony structures (primary) Orbital soft tissues (inferior to MRI)	Orbital soft tissues
Disadvantages	High operator-dependence Limited to superficial structures Reliance on single parameters	Ionizing radiation exposure Poor soft tissue contrast resolution	Long acquisition time Contraindications (metallic implants, claustrophobia, etc.) Relatively limited accessibility
Advantages	Non-invasive Absence of ionizing radiation High accessibility and portability	Clear visualization of morphological alterations	Multi-parametric sequences (T1WI, T2WI, STIR, DWI) Comprehensive tissue characterization (edema, fibrosis, fat proliferation)
Cost	Price-friendly	Medium	Expensive
Diagnosis	Not recommended	Recommended	Recommended
Activity Assessment Capability	Limited	Limited	Excellent (Multi-tissue evaluation, comprehensive quantitative parameters)
Capability in Predicting DON	Weak	Medium (Based on morphology, e.g., orbital apex crowding index)	Strong (Early identification via combined morphological and cellular damage assessment)
Role in Therapeutic Response Evaluation	Limited application	Commonly used	Widely applied in clinical practice and research
Primary Clinical Indications	Initial screening	Preoperative and postoperative assessments, Disease staging, exclusion of other masses	Comprehensive disease assessment, DON prediction, Drug and surgical therapy monitoring
Moderate patients	Secondary	Necessary (if corneal and optic nerve involvement, patients need surgery as soon as possible)	Priority

## 2 Application of ultrasonography in TAO

Color Doppler Imaging (CDI), a safe and non-invasive tool, allows real-time evaluation of hemodynamic features. In patients with active TAO, inflammation can cause endothelial cell damage (3), which inhibits vasoconstriction, leading to the dilation of orbital blood vessels and increased blood flow. Ultrasonography evaluation of the blood flow characteristics in the superior ophthalmic vein and the ophthalmic artery provides critical evidence for the diagnosis and assessment of TAO.

Research indicates that enlargement of the extraocular muscles in TAO patients may compress the orbital veins, resulting in decreased maximum blood flow velocity and volume in the superior ophthalmic vein compared to normal controls (4, 5). Notably, a minimum blood flow velocity of 3.99 cm/s in the superior ophthalmic vein serves as a significant marker for distinguishing between active and inactive TAO, with a diagnostic sensitivity of 91.2% and specificity of 81.2% (4). Furthermore, severe orbital venous stasis is strongly associated with the onset of DON, highlighting the potential utility of assessing superior ophthalmic vein blood flow in predicting the development of DON. In terms of arterial blood flow, the end-diastolic velocity of the ophthalmic artery in patients with TAO is significantly elevated compared to normal controls (4, 6), while the ophthalmic artery resistance index is reduced relative to normal controls (4). This resistance index serves as an indicator for evaluating changes in the condition before and after surgical treatment (7).

Although ultrasonography offers advantages such as rapidity, non-invasiveness, and cost-effectiveness, its accuracy and generalizability in clinical applications are constrained by factors such as vascular diameter, resistance, impedance, compliance, equipment performance, and patient cooperation.

## 3 Application of CT in TAO

CT has recognized advantages in bone structure imaging. It is an important evaluation method for patients with TAO before undergoing orbital decompression surgery, and provides a key basis for the operator to formulate the operation plan and determine the decompression range. In addition, with the development of imaging technology, CT has been gradually applied to the quantitative measurement of soft tissues such as extraocular muscles, intra-orbital fat tissue and lacrimal glands, providing objective indicators for diagnosis and assessment.

### 3.1 Application of CT in quantitative measurement of TAO

The extensive utilization of CT in evaluating TAO has introduced a novel approach for quantifying exophthalmos. In comparison to the conventional Hertel exophthalmometer, CT-based exophthalmometry not only broadens the scope of applicability but also exhibits notable advantages in terms of accuracy and objectivity (8–10). Nevertheless, the potential risk of ionizing radiation associated with CT constrains its extensive use in exophthalmos measurement. Quantitative assessments derived from CT imaging also facilitate the identification of potential imaging biomarkers and their association with clinical characteristics (11). Research indicates a positive correlation between the volume of extraocular muscles and the severity of TAO, with a marked increase observed progressively from healthy individuals to those with mild and severe TAO (12). Moreover, parameters associated with extraocular muscles (EOMs) exhibit significant correlations with visual function impairment and ocular motility deficits in patients with TAO (13). The quantitative assessment of EOMs and adipose tissue using CT is essential for the classification of TAO. Clinically, fat-dominant TAO is typically characterized by upper eyelid retraction, lower eyelid retraction, and exophthalmos, while muscle-dominant TAO is primarily associated with restricted ocular motility, diplopia, and strabismus (14). These distinct subtypes of TAO may be linked to different differentiation mechanisms of orbital fibroblasts (15).

### 3.2 Application of CT in diagnosis and prediction of TAO

The benefits of quantitative measurements using CT are further evidenced in the diagnostic and prognostic capabilities for TAO. Studies suggest that hypertrophy of the retro-orbicularis oculi fat and sub-orbicularis oculi fat may possess significant diagnostic value (16). Furthermore, volume-related parameters demonstrate high precision in predicting the severity of TAO, with an accuracy rate of 0.838 and an area under the curve (AUC) of 0.929 (12). Regression models developed based on parameters such as the total volume of extraocular muscles, lacrimal gland volume, intraorbital fat volume, and lacrimal gland density also exhibit significant diagnostic value in evaluating TAO activity (17). An observational study comprising 50 control subjects and 50 patients diagnosed with TAO has demonstrated that the extraocular muscle index is a reliable predictor of the overall inflammatory status in patients with TAO (18).

### 3.3 Application of CT in diagnosis of DON

DON is one of the most severe complications of TAO, and its clinical diagnosis lacks a standardized criteria, primarily relying on nonspecific symptoms such as vision loss, visual field defects, and color vision impairment (19, 20). Although the precise mechanism of DON is not fully elucidated, it is currently believed that mechanical compression of the optic nerve by orbital tissues, including extraocular muscles and intraorbital fat, is a major contributing factor. Statistics indicate that over 90% of DON cases are associated with optic nerve compression due to extraocular muscle hypertrophy (21). Consequently, improving the accuracy of early DON diagnosis is a clinical priority. CT imaging parameters are crucial in diagnosing DON. Current research highlights that optic nerve crowding (*P*<0.001) and intracranial fat prolapse (*P*<0.05) serve as independent risk factors for concurrent optic neuropathy in patients with TAO (22). Traditionally, the Barrett index has been utilized as a diagnostic indicator, demonstrating a sensitivity of 79% and a specificity of 72% (23). Among the volumes of individual extraocular muscles, the medial rectus volume emerges as the most robust predictor of DON, with a sensitivity of 73.7% and a specificity of 86.7% (24). The volumetric orbital apex crowding index (VACI) exhibits superior diagnostic efficacy, with a sensitivity of 92%, specificity of 86%, and an accuracy of 88% (25). Furthermore, the combined use of VACI and thyrotropin receptor antibodies (TRAb) levels may enhance the predictive accuracy for DON (26). A retrospective study further illustrated the exceptional diagnostic efficacy for detecting DON through the application of machine-learning radiomics analysis on CT scans (27).

### 3.4 Application of CT in efficacy evaluation of TAO

The condition of TAO can be assessed through changes observed in exophthalmos and parameters related to the extraocular muscles and orbital fat, as measured by CT. This imaging modality has become increasingly utilized for evaluating treatment efficacy and optimizing therapeutic strategies (28–31). For example, the diameter of the extraocular muscles and the muscle diameter index may serve as predictors for the effect of extraocular muscle contraction following retrobulbar glucocorticoid injections (32). This highlights the significant clinical importance of quantitative CT analysis in the personalized treatment of TAO.

In conclusion, CT was utilized to assess alterations in orbital structure, thereby providing essential support in the diagnosis of TAO and its complications, and offering a reliable foundation for clinical decision-making.

## 4 Application of MRI in TAO

In comparison to ultrasound and CT, MRI offers unparalleled value in both clinical research and practical applications related to TAO. This is attributed to its superior soft tissue resolution, multiparametric imaging capabilities, and lack of ionizing radiation. MRI facilitates precise visualization of morphological changes in structures such as extraocular muscles, adipose tissue, lacrimal glands, and the optic nerve. Additionally, it allows for a comprehensive evaluation of pathological characteristics in affected tissues through multi-sequence and multiparametric imaging techniques (33). These advantages position MRI as an objective foundation for accurate diagnosis, assessment of disease severity, and monitoring of therapeutic interventions in the management of TAO.

### 4.1 Multimodal application of MRI in the evaluation of extraocular muscles in TAO

Multimodal MRI technology offers a distinctive approach for the comprehensive evaluation of extraocular muscles involvement in TAO by integrating structural, functional, and quantitative imaging modalities. This technology not only diagnoses and assesses disease severity through structural parameters such as extraocular muscles cross-sectional area and volume (34), but also facilitates the monitoring of disease progression via functional imaging and quantitative analyses. While conventional T2-weighted imaging (T2WI) does not reveal significant differences in extraocular muscles hyperintensity between active and inactive TAO patients (35, 36), the signal intensity ratio of extraocular muscles to ipsilateral temporal muscle [SIR (EOM/temporalis)] or SIR of EOM to ipsilateral cerebral white matter demonstrates a correlation with the CAS, thereby providing additional evidence for disease staging (37–39).

Dynamic contrast-enhanced MRI (DCE-MRI) is utilized to quantify tissue microcirculation, with its parameters serving as reliable biomarkers for evaluating disease activity (40). Research indicates distinct microcirculatory characteristics in extraocular muscles across different stages of TAO (41, 42). Several DCE-MRI parameters exhibit significant correlations with the CAS and demonstrate positive predictive values of approximately 90% for disease activity (35). Hu’s study further substantiates the clinical utility of DCE-MRI in monitoring TAO (43). Diffusion-weighted imaging (DWI) assesses restricted water diffusion in extraocular muscles via the apparent diffusion coefficient (ADC), facilitating the early detection of inflammatory changes during asymptomatic phases (44). Patients with TAO show significantly elevated mean ADC values in extraocular muscles compared to healthy controls (45), with ADC values in the medial, inferior, and lateral rectus muscles positively correlating with inflammatory subscores in the VISA classification system (46). Nonetheless, the association between ADC values and CAS remains a subject of debate in the literature (44, 45). Diffusion tensor imaging (DTI) serves as an important instrument in evaluating the disease activity associated with TAO (47).

Quantitative imaging modalities, such as T2 mapping, facilitate the sensitive detection of early extraocular muscle involvement (48). T1 mapping further elucidates fat infiltration patterns that are strongly correlated with refractory diplopia, demonstrating high diagnostic accuracy (AUC=0.89) in patients with TAO who have a history of diplopia resolution (49). The extracellular volume (ECV) functions as an objective quantitative biomarker for evaluating the extent of extraocular muscle fibrosis (50).

The integrative application of multimodal MRI technologies significantly enhances diagnostic precision and dynamic monitoring capabilities for TAO-related extraocular muscle pathology, thereby refining the comprehensive MRI-based evaluation framework for the pathological progression of TAO.

### 4.2 Applications of MRI in the assessment of orbital adipose tissue and exophthalmos in TAO

Orbital fibroblasts exhibit the potential for adipogenic differentiation, and the subsequent expansion of adipose tissue volume is strongly correlated with the presence of exophthalmos. Quantitative MRI analyses have demonstrated that both total orbital adipose tissue volume (*r*=0.70, *P*=0.0006) and anterior orbital adipose tissue volume (*r*=0.64, *P*=0.0023) show significantly stronger correlations with the severity of exophthalmos compared to extraocular muscle volume (*r*=0.58, *P*=0.008). This suggests that adipose hyperplasia is a key factor in the progression of proptosis (51). Consequently, therapies targeting adipose metabolic pathways may represent promising new strategies for mitigating proptosis in patients with TAO. Nevertheless, no significant associations were found between orbital adipose volume and variables such as patient age, disease duration, or CAS (52).

### 4.3 Applications of MRI in the assessment of lacrimal gland in TAO

Patients with TAO often exhibit symptoms indicative of lacrimal gland dysfunction, such as dry eyes and conjunctival hyperemia. Recent research has increasingly identified the lacrimal gland as a significant target organ in TAO. The expression of thyroid-stimulating hormone receptor (TSHR) by lacrimal gland acinar cells suggests that autoimmune responses in TAO may lead to damage of the lacrimal gland (53). Studies have demonstrated that patients with TAO not only experience altered tear composition (54–56), but also exhibit increased lacrimal gland volumes (57, 58). Although approximately 30% of TAO patients present with lacrimal gland enlargement (59), no relevant morphological parameters of lacrimal gland have been identified as effective in differentiating between active and inactive TAO (57, 60, 61). Further investigations have shown that the extent of lacrimal gland protrusion serves as a critical imaging marker for differentiating disease stages in TAO. Patients with active TAO exhibit significantly greater lacrimal gland protrusion compared to those with inactive disease, with a linear correlation observed with TRAb levels (62). Moreover, the degree of lacrimal gland prolapse positively correlates with the CAS, proptosis, and extraocular muscle volume, providing an additional indicator for assessing TAO activity (63).

Functional imaging modalities are instrumental in assessing disease activity in TAO. DTI has revealed significant variations in the ADC values and fractional anisotropy (FA) of the lacrimal gland when comparing TAO patients to healthy controls, as well as between active and inactive TAO subgroups (64). The combination of lacrimal gland T2 mapping values with clinical parameters has the potential to optimize the CAS system (65). Furthermore, quantitative MRI parameters are capable of detecting fat infiltration and fibrosis within the lacrimal gland. Wu et al. demonstrated that certain inflammatory and fibrosis-related markers in the lacrimal gland are markedly elevated in TAO patients relative to those with Graves’ disease, facilitating the differentiation between TAO and Graves’ disease (66). These findings offer novel insights into the pathological mechanisms underlying TAO and provide a foundation for the development of personalized therapeutic strategies.

### 4.4 Multimodal application of MRI in the evaluation of DON

MRI exhibits significant technical advantages in the evaluation of DON, offering essential imaging support for early diagnosis and precise therapeutic intervention. The Dixon- T2WI technique effectively suppresses the interference of orbital fat signals, thereby enhancing the clarity of optic nerve visualization and minimizing artifacts, demonstrating superior sensitivity and specificity compared to regular MRI sequences (67). Functional imaging provides further insights into the pathological mechanisms underlying DON. Reduced ADC values in the optic nerve indicate secondary ischemic alterations due to mechanical compression (68). DTI reveals significantly increased axial diffusivity (AD), radial diffusivity (RD), and mean diffusivity (MD) (*P*=0.003-0.033), along with decreased FA (*P*=0.018), quantitatively indicating disruption of optic nerve axonal integrity and glial cell damage. Additionally, DTI demonstrates robust diagnostic performance, with an AUC of 0.801 (69). Another study also verified the above view (70).

The integration of multimodal MRI has markedly improved diagnostic accuracy. Specifically, the SIR of the optic nerve 3mm posterior to the globe and the orbital apex extraocular muscle index have been identified as independent risk predictors for DON. A combined model incorporating these parameters exhibited excellent diagnostic performance, with an AUC of 0.943 (71), thereby providing an objective basis for the early identification of high-risk patients. Additionally, studies have indicated potential microstructural alterations within the visual pathway of patients with DON. Further integration of orbital MRI with diffusion kurtosis imaging (DKI) for the assessment of the intracranial visual pathway could significantly enhance comprehensive diagnostic capabilities (68, 72). These investigations not only advance the understanding of the mechanisms underlying optic nerve injury but also offer critical references for the early detection and intervention of optic neuropathies.

### 4.5 Application of MRI in efficacy evaluation of TAO

MRI has been extensively utilized in clinical and scientific research to assess therapeutic efficacy. MRI-based assessments of morphological metrics in extraocular muscles and adipose tissue serve as valuable tools for evaluating treatment responsiveness (73). The study further corroborated the effectiveness of corticosteroid therapy in conjunction with orbital radiotherapy in Asian patients with active moderate-to-severe TAO (73). Moreover, the SIR of the levator palpebrae muscle has shown predictive utility for the efficacy of triamcinolone acetonide injections, achieving a sensitivity of 87.5%, specificity of 66.7%, and an AUC of 0.840 (74). The combination of extraocular muscle SIR with serum lipid metabolism parameters may enhance the prediction of responses to glucocorticoid (GC) therapy in patients with active and moderate-to-severe TAO (75). A retrospective study has also identified that the percentage change in SIR(EOM/temporalis)_MAX_ following tocilizumab treatment may serve as a predictive indicator for the necessity of surgical intervention in patients with hormone-resistant DON (76). Additionally, DCE-MRI parameters reveal significant distinctions between responders and non-responders to GC therapy in active TAO, providing crucial insights into the effects of TAO-related microcirculatory alterations on treatment outcomes (43, 77). Despite the limited sample size in some studies, these research findings offer a scientific foundation for developing individualized treatment strategies. Given the characteristics of MRI, which include multiple sequences and parameters, MRI holds greater significance than CT in optimizing the therapeutic efficacy of TAO.

## 5 Conclusion

Imaging modalities are integral to the disease assessment, therapeutic monitoring, and early diagnosis of DON in TAO. Techniques such as ultrasound, CT, and MRI each offer distinct advantages in assessing hemodynamic changes, extraocular muscles, adipose tissue, and the optic nerve. MRI, in particular, not only provides detailed visualization of orbital soft tissue structures through conventional sequences but also utilizes functional imaging techniques and quantitative analyses to evaluate pathological changes such as tissue edema, fat infiltration, and fibrosis. This capability facilitates the identification of diverse imaging biomarkers crucial for disease staging and treatment monitoring. Recent advancements in radiomics have markedly advanced research in the diagnosis and management of TAO (78–80). Additionally, emerging neuroimaging studies have revealed altered functional activity in brain regions, including the insular cortex, inferior temporal gyrus, and superior frontal gyrus, in patients with TAO. These changes may be associated with visual function and peripheral immune status (81–84). These findings provide a foundation for further exploration of the mechanisms underlying TAO and DON.

Nevertheless, the clinical application of imaging technologies encounters several challenges. The prolonged duration of MRI scans, coupled with their high cost, restricts their accessibility for primary care and dynamic monitoring purposes. Additionally, certain functional imaging parameters necessitate validation in larger cohorts. Future research should prioritize the comprehensive exploration and validation of imaging techniques within larger populations to fully realize their potential in the early diagnosis of TAO, personalized treatment, and mechanistic investigations. This approach aims to ultimately offer comprehensive and effective support for clinical practice.
